# Cell-based therapy in prophylaxis and treatment of chronic graft-versus-host disease

**DOI:** 10.3389/fimmu.2022.1045168

**Published:** 2022-11-17

**Authors:** Matteo Doglio, Rachel E. Crossland, Ana C. Alho, Olaf Penack, Anne M. Dickinson, Georg Stary, João F. Lacerda, Günther Eissner, Marit Inngjerdingen

**Affiliations:** ^1^ Experimental Haematology Unit, Division of Immunology Transplantation and Infectious Diseases, Vita-Salute San Raffaele University, Milan, Italy; ^2^ Translational and Clinical Research Institute, Faculty of Medical Sciences, Newcastle University, Newcastle upon Tyne, United Kingdom; ^3^ JLacerda Lab, Hematology and Transplantation Immunology, Instituto de Medicina Molecular João Lobo Antunes, Faculdade de Medicina da Universidade de Lisboa, Lisbon, Portugal; ^4^ Serviço de Hematologia e Transplantação de Medula, Hospital de Santa Maria, Centro Hospitalar Universitário de Lisboa Norte, Lisbon, Portugal; ^5^ Department of Hematology, Oncology, and Cancer Immunology, Charité Universitätsmedizin Berlin, Berlin, Germany; ^6^ Alcyomics Ltd, Newcastle upon Tyne, United Kingdom; ^7^ Department of Dermatology, Medical University of Vienna, Vienna, Austria; ^8^ Ludwig Boltzmann Institute for Rare and Undiagnosed Diseases, Vienna, Austria; ^9^ CeMM Research Center for Molecular Medicine of the Austrian Academy of Sciences, Vienna, Austria; ^10^ Systems Biology Ireland, School of Medicine, Conway Institute, University College Dublin, Dublin, Ireland; ^11^ Department of Pharmacology, University of Oslo and Oslo University Hospital, Oslo, Norway

**Keywords:** chronic GVHD, Tregs, CAR, NK cells, ILCs, MSCs, extracellular vesicles

## Abstract

Hematopoietic allogeneic stem cell transplantation (allo-SCT) is a curative option for patients with hematological malignancies. However, due to disparities in major and minor histocompatibility antigens between donor and recipient, severe inflammatory complications can occur, among which chronic graft-versus-host disease (cGVHD) can be life-threatening. A classical therapeutic approach to the prevention and treatment of cGVHD has been broad immunosuppression, but more recently adjuvant immunotherapies have been tested. This review summarizes and discusses immunomodulatory approaches with T cells, including chimeric antigen receptor (CAR) and regulatory T cells, with natural killer (NK) cells and innate lymphoid cells (ILCs), and finally with mesenchymal stromal cells (MSC) and extracellular vesicles thereof. Clinical studies and pre-clinical research results are presented likewise.

## 1 Introduction

**Figure 1:**
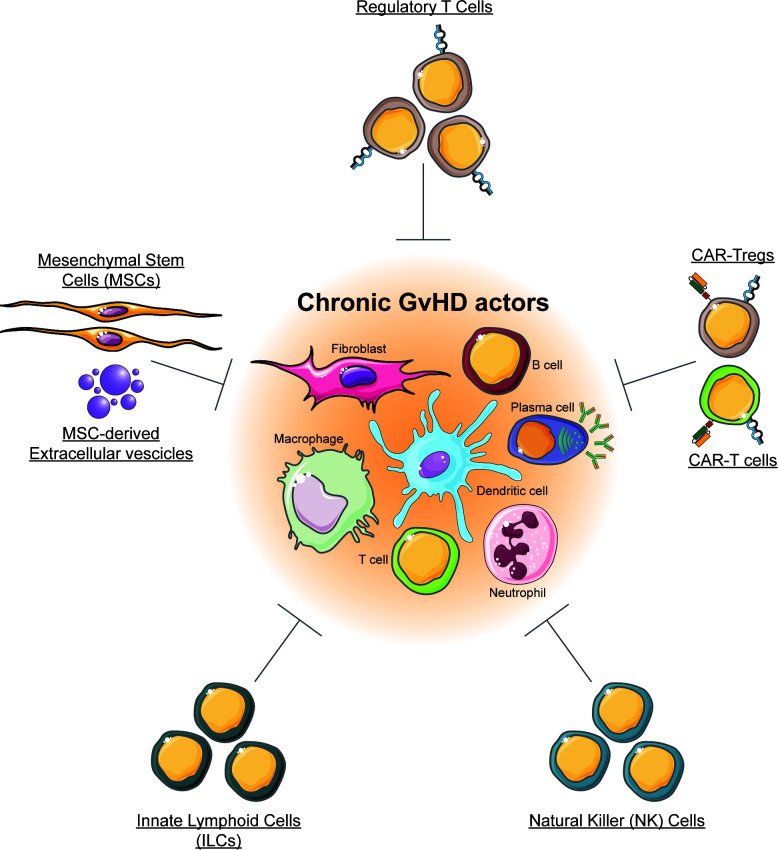
Cellular therapy for chronic GVHD. The figure illustrates cell types that are currently exploited or that can be exploited for chronic GVHD, and that are discussed in this review.

Chronic graft-versus-host disease (cGVHD) is a multisystem inflammatory disease that results from a multifaceted response of allogeneic immune effector cells against a variety of tissues in patients surviving early phases of allogeneic hematopoietic stem cell transplantation (alloHSCT). Mild forms of cGVHD associate with better control of the malignant disease. In contrast, severe cGVHD is the main reason for morbidity and mortality in long-term survivors of alloHSCT. Standard therapies for severe cGVHD have limited efficacy and significant toxicity (1), and require suppression of the immune system that may result in severe and/or fatal infections (2). Furthermore, current pharmacological interventions are largely unsuccessful in reversing established fibrosis in cGVHD, without compromising immune function. Therefore, new approaches for the prevention and treatment of cGVHD are needed in order to improve the long-term outcomes and quality of life post alloHSCT (3). Here, we highlight novel strategies to prevent and treat cGVHD using cellular therapy, and discuss the latest developments in this field (**Figure 1**).

Regulatory T cells (Tregs) represent a subset of CD4^+^ T lymphocytes specialized in controlling immune responses and in maintaining immune tolerance. The efficacy of Tregs in GVHD treatment has been initially demonstrated in murine models (4), and further in human patients (5). An innovative possibility to increase the anti-GVHD efficacy of cellular therapy is represented by Chimeric Antigen Receptors (CARs). As an example, CAR-Tregs targeting allo-activated immune cells expressing CD38 were highly effective in reducing GVHD (6). Invariant NKT (iNKT) cells can dampen cGVHD through induction of Tregs. Also, NK cells may be exploited to target the alloreactive cells, while innate lymphoid cells (ILC) that have a crucial role in tissue regeneration of the intestinal tract, could represent an attractive treatment option for severe cases of intestinal GVHD (7). Another focus is treatment with cell types that are involved in tissue homeostasis and repair, as mesenchymal stromal cells (MSCs), and we also discuss the utility of extracellular vesicles derived from MSCs.

## 2 T cell-based therapies

T lymphocytes have a pivotal role in the immune system. Conventional CD4^+^ T cells (Tconvs) secrete a number of immunoregulatory cytokines, while cytotoxic CD8^+^ T cells are equipped to kill malignant or infected cells. T cells have been extensively exploited in the context of bone marrow transplantation, with the aim to recognize and kill tumor cells (Graft-*versus*-Leukemia effect, GvL), and control infections, especially opportunistic ones (8).

However, T cells are also involved in the development of GVHD due to mismatches of HLA class I molecules and minor histocompatibility antigens between the host and the donor, which elicit an inflammatory response (9, 10). For this reason, different strategies have been developed to dampen T cell activation, aiming at blocking this detrimental process (11). Several drugs have been approved so far for the treatment of GVHD, however, their activity is broad and unspecific, acting on different components of the inflammatory cascade with a generalized immunosuppressive action, blocking not only harmful T cells but also vital anti-viral and anti-tumor effects (11, 12).

For this reason, the development of more specific treatments that target directly key components of GVHD, is required. To this end, the same Tconvs could be employed to eliminate specific cell populations that sustain the inflammatory process (13, 14). However, this approach is limited by the low number of antigen-specific cells able to kill relevant pathologic cells (15). A possible solution to this problem is represented by CAR-T-cells.

### 2.1 CAR-T-cells

**Figure 2:**
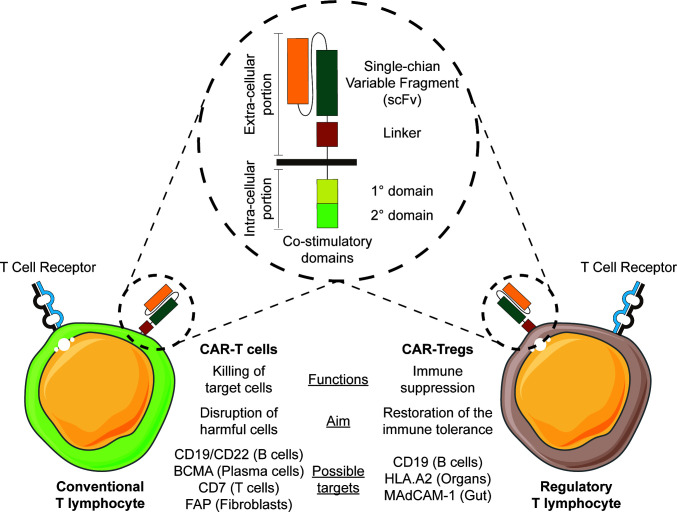
Exploiting CAR-T cell therapy for chronic GVHD. The engineering of CAR constructs (illustrated in the circle) in conventional T cells or T regulatory cells may be exploited in therapy for chronic GVHD.

CARs are chimeric molecules composed of two major components (**Figure 2**). An extracellular part consisting of a single-chain variable fragment (scFv) conferring antigen specificity, is fused to an intracellular part providing the machinery for cell signaling transduction. The first generation of CARs was composed of an intracellular CD3 zeta chain, but this version showed only minimal functionality. In the subsequent generations, different stimulatory domains were fused together, obtaining much higher cellular activation. CARs greatly enhance cellular functions such as cytotoxicity, proliferation, survival and cytokine secretion. The constructs can be inserted in different effector cells, as T cells and NK cells. Their major field of application is in oncology, where CAR-T cells have displayed striking results, in particular against CD19^+^ acute lymphoblastic leukemia (ALL) and B cell-derived lymphomas (16).

While CAR constructs initially were developed for use in cancer therapy (17, 18), some CARs might also be employed to tackle cells that mediate the inflammatory processes occurring during cGVHD (19). Various immune cells contribute to the pathology of cGVHD, and among them B cells produce antibodies and release pro-inflammatory cytokines, and operate as antigen-presenting cells to promote T cell activation. The B cells appear to be hyper-responsive due to both intrinsic and extrinsic factors, favoring the selection of potential harmful cells reactive against both allo- and auto-antigens (20, 21).

Due to their pro-inflammatory properties, the selective depletion of B cells might represent a potential therapeutic option in patients with cGVHD. Several drugs have been approved for this purpose, with variable results. One of the major limitations of the drugs resides in the absence of a clear specificity, often targeting markers that are expressed on multiple cell populations (22). A possible solution is the employment of CAR-T cells, such as the anti-CD19 or the anti-CD22 CARs, which could eliminate B lymphocytes. In particular, a beneficial anti-inflammatory activity of anti-CD19 CAR-T cells have been shown in the context of autoimmune diseases, like systemic lupus erythematosus, where reduced levels of auto-antibodies and immune complexes were demonstrated in lupus-prone mice. However, clinical evidence for a role of anti-CD19 CAR-T cells in the treatment of cGVHD in humans is still lacking (23).

It has been demonstrated that cGVHD patients may develop auto-antibodies during the course of the disease, which might have a role in mediating tissue injuries (24, 25). Pre-clinical mouse models clearly showed that the depletion of antibody-secreting cells could attenuate clinical manifestations of cGVHD (26). Anti-BCMA CAR-T cells can efficiently kill plasma cells and they have been employed for the treatment of patients with multiple myeloma with encouraging results (27). Considering their potential role in the pathogenesis of cGVHD, anti-BCMA CAR-T cells might open new therapeutic scenarios due to the depletion of allo- and auto-reactive plasma cells.

Anti-CD19 and anti-BCMA CARs eliminate B cells and plasma cells, respectively, without a specific activity against the pathological clones. In 2016, Ellebrecht et al. developed a new chimeric construct to selectively target and deplete antigen-specific B cells, called chimeric auto-antibody receptor (CAAR) (28). The authors employed CAAR-T cells for the treatment of pemphigus vulgaris, an autoimmune disease caused by the production of auto-antibodies against the dermal basal membrane, by redirecting T lymphocytes against anti-desmoglein 3 self-reactive B cells, showing encouraging results in pre-clinical mouse models. With this approach, the authors managed to selectively deplete only self-reactive cells. CAARs might be employed in the context of cGVHD to selectively eliminate those cells that sustain the pathologic process, avoiding the generalized depletion of either B lymphocytes or plasma cells caused by anti-CD19 and anti-BCMA CAR-T cells (28).

Beside B lymphocytes, also T cells have an important role in the pathogenesis of GVHD, being able to recognize HLA mismatches and to stimulate pro-inflammatory responses. Several drugs target T cells, controlling the inflammatory response and showing encouraging results in cGVHD patients. Also, CAR-T cells might be employed to this extent: anti-CD7 CAR-T cells have been designed to specifically eliminate T cells in the context of T cell malignancies, due to the selective expression of CD7 on T lymphocytes and NK cells. To circumvent a potential fratricide effect, engineered cells were manipulated with CRISPR/Cas9 to disrupt the CD7 gene. This product showed promising result in cancer and might be potentially employed also in the context of cGVHD to control the inflammatory response mediated by T cells (29).

Apart from immune cells, other cells participate in the generation of tissue injuries in cGVHD. In particular, fibroblasts have a well-established role in mediating collagen deposition and fibrosis, especially in the skin and lungs. Lung fibrosis represents one of the most important causes of disability and mortality in cGVHD patients. Macrophages and T cells have an important role in inducing fibroblast proliferation and collagen deposition and their depletion ameliorates the occurrence of fibrosis in pre-clinical models. In particular, the polarization of macrophages toward the M2 phenotype is a key event in this process: these cells secrete high amounts of TGF-β and IL-4 that stimulate fibroblasts and boost their activity (30, 31). In 2019, Aghajanian and colleagues developed an anti-fibroblast activation protein (FAP) CAR construct to target and kill fibroblasts (32). Anti-FAP CAR-T cells were evaluated in a pre-clinical mouse model of cardiac fibrosis, proving effective in ameliorating the cardiac lesions. In particular, treated mice displayed a significant reduction of the number of fibroblasts and consequently of the fibrosis compared to the control group. The therapy was coupled to an improvement of cardiac functional parameters, like fractional shortening and mitral valve inflow velocity (32). Considering the important role of fibroblasts in cGVHD pathogenesis, anti-FAP CAR-T cells might represent a promising therapeutic tool for patients in order to limit fibroblast proliferation and subsequent fibrosis, especially in lungs.

### 2.2 Regulatory T cells (Tregs)

Tregs represent a subset of CD4^+^ T lymphocytes specialized in controlling immune responses and in maintaining immune tolerance (33). Tregs can be divided into two distinct subsets: thymic or centrally derived Tregs, and peripherally induced Tregs. The first group encompasses those cells that directly exit the thymus with already acquired immune suppressive capabilities, and are responsible for the maintenance of self-tolerance. The second group includes those lymphocytes generated in the periphery after the activation of CD4^+^ Tconvs and are thought to control local activation of the immune response (34, 35). A unique marker for the identification of Tregs is still lacking, and up to now a combination of markers is required for their identification. Tregs are characterized by high expression of the FoxP3 (Forkhead Box P3) transcription factor, which represents the master regulator for Treg development and function. High CD25 (the alpha subunit of the interleukin-2 (IL-2) receptor) expression level is another Treg feature, also denoting their great dependency of IL-2 (36).

Regardless of their origin, Tregs are endowed with suppressive capabilities that can be exerted either *via* the secretion of mediators or *via* cell-to-cell contact. Their action is fundamental for the correct homeostasis of the immune system. Indeed, patients diagnosed with the IPEX syndrome (congenital deficiency of FoxP3) lack functional Tregs and suffer from several autoimmune manifestations (37).

In the context of HSCT, the equilibrium between effector and suppressive T cells appears to play an important role in the development of cGVHD. Even though some studies did not find lower Treg function in these patients, a plethora of studies have now shown that altered Treg homeostasis and decreased Treg counts are associated with cGVHD (38–43).

Based on the recognition that Treg counts are decreased in patients with cGVHD and that the emergence of this syndrome is associated with poor immune tolerance post-transplant, Koreth et al. at the Dana-Farber Cancer Institute conducted Phase 1 and Phase 2 studies investigating whether daily low-dose IL-2 administered sub-cutaneously for 8 to 12 weeks would have an effect in patients with steroid refractory (SR) cGVHD, and if there was any correlation with Treg counts and function. In these trials, approximately 50-60% of the patients responded clinically, some quite significantly, allowing for steroid doses to be tapered, which was correlated with a significant increase in Treg counts (44, 45). These studies set the stage for trials of donor Treg infusions, with the rationale that the patients not responding to low-dose IL-2 were likely to have an exhausted Treg compartment and could therefore benefit from the infusion of fresh donor Tregs, with or without low-dose IL-2.

#### 2.2.1 cGVHD prevention employing Tregs

Several preclinical and clinical studies have suggested that enriching donor grafts with donor Tregs could improve the outcome of transplant by facilitating immune reconstitution and reducing the risk of GVHD, without interfering with the anti-tumor activity (4, 46, 47).

A major advantage of Treg-therapy is the capacity to induce allo-specific suppression, avoiding the side effects of general immunosuppressive drugs. In humans, Treg-based cell therapy consists of isolation and purification of circulating Tregs from peripheral blood (PB) or umbilical cord-blood (UCB), expansion, and transfer to the patient. However, the purification and expansion of sufficient numbers of Tregs for effective immunotherapy is hampered by their low number in peripheral blood, the lack of specific phenotypic markers for their isolation, and the slow rate of *in vitro* expansion for clinical use (48). To bypass these issues, several protocols have been applied for *de novo* generation and expansion of functional Tregs (49, 50).

Expansion protocols usually start from peripheral blood mononuclear cells (PBMCs), either involving the use of stimulatory antibodies (e.g. anti-CD3, soluble CD28, anti-CD3/CD28 magnetic beads) or engineered APCs. In addition, several compounds have been employed to enrich for Tregs at the end of the cell culture, either by blocking Tconv activation, boosting Treg expansion or combining the two approaches (51). Tregs can be isolated from PBMCs using a CD4^+^CD25^+^CD127^dim/−^ Treg cell isolation kit. However, activated Tconvs display high levels of CD25 and up-regulate FoxP3, thus limiting the sorting strategy. Furthermore, experimental evidence showed that cells with a Treg phenotype in pro-inflammatory environments can be reprogrammed toward Th17 pro-inflammatory cells with an amplification rather than an attenuation of tissue damage. Considering these factors, the risk of having a contaminant Tconv population after sorting for Tregs is high, in particular in *in vitro* expanded cells starting from PBMCs (52, 53).

In the last decade, the safety and feasibility of Treg-based cell therapy for GVHD prevention and treatment has been tested in several clinical trials, as mentioned in **Table 1**. The Perugia group was the first to provide proof of principle that purified natural polyclonal Tregs can prevent GVHD mediated by Tconvs in the setting of haploidentical transplantation for hematologic malignancies, with no pharmacologic GVHD prophylaxis. Patients received CliniMACS immunomagnetic purified donor Tregs (2×10^6^/kg) at day -4, followed by a purified CD34^+^ hematopoietic stem cell graft and Tconv infusion at day 0. The upfront infusion of Tregs into conditioned patients permitted the *in vivo* expansion of Tregs, as previously described in mouse models (66). To establish the optimal Treg : Tconv ratio, different escalating doses of donor Tconv (0.5 - 2x10^6^/kg) were administered. Di Ianni et al. reported the first results from 28 patients (24 in any complete remission and 4 in relapse) showing a rapid immune reconstitution with a wide repertoire, an increased reconstitution of pathogen-specific CD4^+^ and CD8^+^ T cells, and a low incidence of CMV reactivation. Two patients developed ≥ grade 2 acute GVHD (aGVHD) and no patients developed cGVHD (5). Martelli et al. extended the analysis to 43 patients with high-risk acute myeloid leukemia (AML) in any remission to investigate whether Treg-Tconv adoptive immunotherapy would prevent post-transplant leukemia relapse. In this setting, the incidence of ≥ grade 2 aGVHD was 15% and only one patient developed cGVHD (54). These results show that adoptive therapy with Tregs is a feasible option in HLA-haploidentical transplant, since it enables the control of alloreactive co-infused Tconvs, with low incidence of GVHD and persistence of a strong GvL effect.

**Table 1:** Principal trials on Treg-cell based therapy in GVHD prophylaxis and treatment.

Design	Indication (population included)	Intervention/Treg mean cell dose	N. ° pts	PI/Location	Status	Reference
Phase I, single center	Prophylaxis (adults with hematologic malignancy)	Donor Treg + Tconv - Haplo SCT (2×10^6^/kg)	28	Di Ianni et al./ Perugia	Completed	(5)
Phase II, single center	Prophylaxis (adults with high risk AML)	Donor Treg + Tconv - Haplo SCT (2×10^6^/kg)	43	Martelli et al./ Perugia	Completed	(54)
Phase II, single center	Prophylaxis (elderly with high risk AML)	Donor Treg + Tconv - Haplo SCT + TMLI (2×10^6^/kg)	14	Pierini et al./ Perugia	Completed	(55)
Phase I, single center	Prophylaxis (adults with hematologic malignancy)	Expanded 3^rd^ party UCB Treg - UCBT (1-30x10^5^/Kg; 3-10x0^6^/Kg)	23;11	Brunstein et al./Minnesota	Completed	(56, 57)
Phase I/II, single center	Prophylaxis (adults with hematologic malignancy)	Expanded and fucosylated 3^rd^ party UCB Treg (1×10^6^/kg)	5	Kellner et al./ Houston	Completed	(58)
Phase I/II, single center	Prophylaxis (adults with hematologic malignancy)	Highly purified Treg + Tconv – MAC (1-3×10^6^/kg)	12	Meyer et al./ Stanford	Completed	(59)
Phase II, single center	Prophylaxis (child, adult, elder high risk AML)	Donor Treg + Tconv - Haplo SCT + TMLI/TBI (2×10^6^/kg)	50	Pierini et al./ Perugia	Completed	(60)
Phase I	Treatment cGvHD	Expanded donor Treg + IL-2 (1x10^5^ Treg/kg)	2	Trzonkowski et al./ Gdańsk,	Completed	(61)
Phase I	Treatment cGvHD	Expanded donor Treg ± IL-2 (2.4x10^6^ Treg/kg)	5	Theil et al./ Dresden	Completed	(62)
Phase I	Treatment cGvHD	Donor Treg (1,5x10^6^ Treg/kg)	10	Johnston et al./Stanford	Completed	(63)
Phase I/II TREGeneration	Treatment cGvHD	Donor Treg (dose escalation; MTD)		Lacerda et al./ Lisboa	Recruiting	–
Phase I TREGeneration	Treatment cGvHD	Donor Treg + IL-2		Koreth et al./ Boston	Completed	(64)
Phase I/II, TREGeneration	Treatment cGvHD	Donor Treg (multiple infusion dose escalation; MTD)		Arpinati et al./ Bologna	Recruiting	–
Phase I/II TREGeneration	Treatment cGvHD	Expanded donor naïve Treg		Edinger et al./Regensburg	Recruiting	–
Phase I/II, TREGeneration	Treatment cGvHD	Donor Treg + rapamacine and/or IL-2		Baron et al./ Liège	Recruiting	–
Phase I TREGeneration	Treatment cGvHD refractory to ruxolitinib	Donor Treg		Perez Simon et al./Seville	Recruiting	–
Phase II, multicenter	Treatment cGvHD	Autologous T-cell depleted + Treg expanded	17	Ahmad et al./Canada	Completed	(65)

More recently, the same Treg-Tconv adoptive immunotherapy protocol was tested in combination with a low-toxicity conditioning regimen including total marrow and lymphoid irradiation (TMLI). Pierini et al. designed this protocol for aged or unfit AML patients, unable to tolerate total body irradiation (TBI). Fourteen patients were included and the results showed that Tregs (2x10^6^/kg, day-4) can prevent cGVHD with no need for post-transplant immunosuppression, and the co-administration of Tconvs (1x10^6^/kg, day-1) could eradicate minimal residual disease and ensure relapse free survival (55). These results have been replicated in an updated trial conducted by the same group, which combined and age-adapted myeloablative conditioning regimen, using TBI or TMLI, with Treg-Tconv adoptive immunotherapy (60). At a median follow-up of 29 months, moderate/severe cGVHD occurred in only one patient and 2 patients relapsed.

Considering the low number of Tregs in the peripheral blood, several groups have explored *ex-vivo* Treg expansion for therapeutic application using different protocols while retaining Treg suppressive activity (67, 68). The first-in-human clinical trial demonstrating safety and preliminary effectiveness of *ex-vivo* expanded Tregs for GVHD prophylaxis in double UCB transplant was published in 2011 by Brunstein et al. (68). Cryopreserved UCB-derived Tregs were expanded in culture with anti-CD3/anti-CD28 antibody-coated beads and recombinant human IL-2. Twenty-three patients received a dose of 0.1-30 ×10^5^ Treg/kg at day +1, followed by GVHD prophylaxis with cyclosporine A (CsA)/sirolimus and mycophenolate mofetil (MMF). After infusion, Tregs could be detected for 14 days, with the greatest proportion of circulating Tregs observed on day + 2. There were no transfusion-related adverse events and compared with identically treated 108 historical controls, there was a reduced incidence of grade II-IV aGVHD (43% vs 61%; p=0.05) with no increased risk of infection, relapse, or early mortality (56).

In 2016 an extended analysis was published, confirming the safety profile and maximal tolerated dose. Here, in order to maximize the infused Treg dose and increase Treg potency, selected CD25^+^ UCB cells were expanded in cultures with K562 cells modified to express the high-affinity Fc receptor CD64 and CD86 (CD28L), in the presence of IL-2. Eleven patients received a dose of 3-100×10^6^ Treg/kg. There were no dose-limiting infusion adverse events. The incidence of grade II-IV aGVHD at 100 days was 9% vs 45% (p=0.05) comparing to a non-randomized Sirolimus/MMF control group. The incidence of cGVHD at one year was 0 vs 14% (57).

Another approach to expanded UCB-derived Tregs for GVHD prophylaxis was developed using fucosylation of the expanded human Tregs in order to improve their trafficking pattern. Tregs bind endothelial E- and P-selectins for trafficking to sites of inflammation, and the incubation of Tregs with fucosyltransferase-VI has shown to effectively increased their homing in severe immunodeficient mice (69). Based on these results, Kellner et al. designed a pilot study of adoptive therapy with fucosylated *ex-vivo* expanded UCB-derived Tregs in allo-HSCT patients. Five patients were included and a dose of 1 x10^6^ Treg/kg was infused at day -1. Although all patients developed grade II-IV aGVHD, there was no evidence of cGVHD at a median follow up of 25 months (58).

Despite the different protocols, the purity of Treg products has been difficult to control, which can limit the efficacy of Treg infusion due to contamination with effector cells. In order to increase Treg purity, Meyer et al. developed a strategy of double Treg selection using immunomagnetic selection and high-speed flow cytometric sorting. This protocol was used in 12 patients with various hematologic malignancies after a myeloablative transplant (59). A purity of 91%-96% was obtained and Tregs were infused on day 0 (1-3x10^6^/Kg) followed by Tconv infusion at 1:1 ratio on day +2. The first 5 patients received cryopreserved Tregs, with 2 patients developing grade II-IV aGVHD. Seven patients received fresh Tregs and single-agent GVHD prophylaxis, with no acute nor cGVHD development at a median of 501 days post HSCT (59). These results suggest that there is a reduced functionality of Tregs after cryopreservation, and that highly purified Tregs may avoid Treg expansion.

#### 2.2.2 Tregs for cGVHD treatment

Although adoptive Treg therapy has been mainly tested in GVHD prevention, ongoing and completed clinical trials investigate the safety and efficacy of polyclonal Treg infusion in the treatment of SR cGVHD. The first-in-human clinical study suggesting Treg infusion as an adjuvant therapy in refractory GVHD was published in 2009 by Trzonkowski et al. (61). Donor-Tregs were incubated with anti-CD3/CD28 beads and expanded with high dose IL-2; the results show significant alleviation of symptoms, allowing reduction of immunosuppression.

In 2015, Theil et al. used adoptive therapy with donor-expanded Tregs to treat refractory cGVHD in patients with AML who underwent allo-HSCT (62). After incubation with anti-CD3/CD28 and expansion with high dose IL-2 and rapamycin for 2–3 weeks, the final products contained mean quantity of 2.4x10^6^ Treg/kg with an average purity of 84.1%. The expanded cells showed suppressive function *in vitro* and were infused at a median time of 35 weeks post-transplant. Five patients were infused; 2/5 patients had an increase of circulating Tregs and partial response of cGVHD. Three patients were alive at the end of the follow-up period and 2 patients developed skin cancers after 4 and 11 months of Treg infusion (62).

Later on, Johnston et al. accessed the safety and tolerability of Treg therapy for SR cGVHD in matched related donor recipients, using highly purified donor-derived Tregs, through clinical scale high speed cell sorting. Tregs were administered in a single infusion, at three different doses. The maximum feasible dose attainable without *ex vivo* expansion was 1.5 x10^6^ Treg/kg, resulting in encouraging preliminary clinical responses (63).

In 2015, the clinical consortium TREGeneration, coordinated by João Lacerda Lab in Lisbon, was created with the primary aim to explore the safety (phase 1) and preliminary efficacy (phase 2) of different donor-derived Treg products for the treatment of patients with SR cGVHD after allo-HSCT. This research is running in parallel in several institutions, using different protocols for Treg preparation and/or administration (**Table 1**). Results from the phase 1 study conducted at the Dana Farber Cancer Institute-Boston were recently published. This group showed that a single infusion of polyclonal Treg-enriched lymphocytes, from the original stem cell donor, followed by daily low-dose IL-2 was safe and well tolerated, with clinical benefit, including in those with inadequate responses to IL-2 alone (64). Furthermore, there was an increase in Treg repertoire diversity, with expansion and long-term persistence of infused Treg clonotypes.

Overall, the safety of Treg adoptive immunotherapy has been successfully validated. However, new questions were raised concerning the ideal source, isolation method, dose and timing of Treg infusion. Ongoing clinical trials investigating the efficacy of Treg infusion for GVHD prevention and treatment in larger populations will help clarify these questions and will give us important additional information about the clinical implementation of these cells in the near future.

#### 2.2.3 CAR-Tregs

Finally, in this new era of gene and cell therapy we anticipate that the use of polyclonal Treg-based therapy will shift towards engineered antigen-specific Tregs, likely able to generate more potent responses, without the concerns of off-target effects (51). In this setting, the development of CAR-Tregs appears to be a promising strategy. In two pre-clinical models, CAR-Tregs demonstrated effectiveness in controlling GVHD manifestations. In 2016, MacDonald et al. generated alloantigen-specific anti-HLA-A2 CAR-Tregs. The authors demonstrated that CAR-Tregs performed better than untransduced Tregs in suppressing the *in vitro* proliferation of HLA-A2 PBMCs. Strikingly, only anti-HLA-A2 CAR-Tregs were able to efficiently prevent the onset of xenogenic GVHD in a mouse model of HSCT (70). Similar results were published in 2017 by Pierini et al. The authors generated customizable CAR-Tregs, specific against CXCL12 and MAdCAM-1 to localize the cells in the gut. Engineered Tregs managed to efficiently suppress the inflammatory response in a mouse model of intestinal acute GVHD, thus ameliorating the clinical picture (71). In addition, Imura et al. in 2020 proved the efficacy of anti-CD19 CAR-Tregs in controlling the clinical manifestations in a mouse model of xeno-GvHD (72).

It is well established that Tregs can preserve the immune tolerance by directly killing activated target cells, e.g. Tconvs and APCs. However, the exact mechanisms are not fully elucidated due to contradictory results, which have been extensively discussed elsewhere (73). A direct cytotoxic effect might represent a potential concern for the use of engineered Tregs, limiting the employment of CAR-Tregs redirected against self-antigens and healthy tissues. Several CAR-Treg studies reported a negligible cytotoxic effect of engineered cells both *in vitro* and *in vivo*. Some authors described a minimal cytotoxicity only *in vitro* culturing the cells at high effector-to-target ratios and only with specific CAR molecules (74–76). It is still not clear whether this might be dependent on the manufacturing protocol, Treg selection, CAR structure or the CAR target. Although a potential cytotoxicity might represent a limitation for the use of anti-self CAR-Tregs, this capacity might be useful in specific circumstances. Indeed, the direct killing of pathogenic cells and the suppression of by-stander ones might help to better control the inflammatory response in GvHD. This is supported by some publications that demonstrated how CAR-Tregs might control GvHD, while preserving the Graft-versus-Tumor (GvT) effect in mice (77). Further studies are required to better elucidate the suppression mechanisms of CAR-Tregs and their regulation.

## 3 NK cells

NK cells are the first lymphocytes to reconstitute after both autologous or allogeneic HSCT, as well as after UCB transplantation. NK cells have been well studied in terms of acute GVHD, where they appear to protect from disease by targeting alloreactive T cells. There are fewer studies on NK cells in the context of cGVHD, and still inconclusive whether they contribute to or protect from disease. As such, there are currently no NK cell-based therapies, but strategies targeting or utilizing NK cells should be explored.

An early study reported that patients with extensive cGVHD had low blood NK cell counts (78), and another study showed that there were fewer NK cells in skin lesions of cGVHD patients compared to aGVHD patients in a cohort of patients receiving allo-HSCT from HLA-matched siblings (79). These finding suggest that low blood NK cell counts may not reflect increased tissue re-localization and infiltration of NK cells into sites of cGVHD. This notion is supported by studies showing correlations of NK cell numbers and their functional capacities with cGVHD outcome. A study comparing UCB with peripheral blood HSCT reported a higher increase of NK cells in recipients of UCB, with concomitant lower rate of cGVHD (80), although the authors could not demonstrate any direct association of risk to NK cell parameters. Another study in pediatric UCB transplant patients, reported that early detection of NK cells with functional capacity was a contributing factor for low incidence of cGVHD (81).

As to any direct role in cGVHD pathology, an early study suggested that NK cells in collaboration with suppressive CD8^+^ T cells could contribute to suppression of autoantibody-producing B cells, and that this function may be compromised in autoimmune diseases (82). Other studies strongly point to involvement of the MICA-NKG2D axis that could be detrimental in terms of cGVHD. Elevated soluble MICA levels in plasma have been associated to cGVHD susceptibility, in particular the MICA-129Val polymorphism (83). The MICA-NKG2D axis is well characterized in the context of cancer, where engagement of MICA on tumor cells by NKG2D unleash NK cell cytotoxicity. Elevated soluble MICA levels in a pro-inflammatory milieu in cGVHD could drive pathogenic IFN-γ from NK cells contributing to exacerbating cGVHD, as suggested in a study by Boukouaci and colleagues (84). Production of INF-γ by donor effector cells associate to onset of cGVHD, and IFN-γ promotes secretion of BAFF from monocytes that promote B cell activation and autoantibody production (85). Thus, dampening IFN-γ production by NK cells through the MICA-NKG2D axis could be a therapeutic option.

A perhaps more feasible avenue is to explore CAR NK cells, in analogy to the use of CAR-T cells described above. Potentially, CAR NK cells targeting CD19 or CD20 could be exploited in cGVHD as a potentially safer alternative to CAR-T cells. The use of CAR NK cells against cancer has shown great promise, and CD19 or CD20 CAR NK-92 cells or allogeneic primary NK cells have successfully been used to eradicate B cell malignancies (86–88). There are currently no trials on CAR NK cells in context of cGVHD, but it is obvious that CAR NK cells could have utility in targeting and eradicating pathologic B cells in cGVHD patients.

## 4 Invariant NKT cells

Invariant NKT cells, also known as classical or type I NKT cells, is a group of T cells with innate properties. The cells express a semi-invariant TCR α-chain (V α 24Jα18 paiting with β11 in humans, or Vα14Jα18 in mice pairing with a restricted set of TCRβ chains (89), together with NK cell receptors. iNKT cells produce high amounts of IL-4 and IFN-γ, and their development is dependent on the transcription factor promyelocytic leukemia zink finger (PLZF). Their TCR recognize glycolipids presented by the non-polymorphic MHC-1-like molecule CD1d (90). Although they are normally in low numbers in peripheral blood, they represent an important population of immunoregulatory cells.

Studies on the relationship of doses of different immune cell populations in allografts have demonstrated that the dose of iNKT cells, as well as Tregs, impact both disease-free survival and overall survival (91). Early studies showed that expanding iNKT cells in murine models of GVHD protected against GVHD through polarization towards a Th2 response and donor Treg expansion, effects that were dependent on IL-4 produced by the iNKT cells (92). Recent studies have demonstrated that iNKT represent a promising candidate for cellular therapy of cGvHD. Adoptive transfer of donor iNKT cells to mice were shown to prevent cGVHD and even reverse lung cGVHD, and the authors suggested this was linked to increased frequencies of follicular Tregs *via* production of IL-4 from the iNKT cells (93). In terms of their future clinical use, several studies have shown the feasibility of expanding iNKT cells using IL-2 and the synthetic ligand α-GalCer (94).

## 5 Innate lymphoid cells

ILCs comprise a group of cells with lymphoid morphology and various functions, ranging from important roles in tissue homeostasis and autoimmunity to immune defense against invading pathogens (95, 96). Depending on the pattern of cytokine secretion and the expression of transcription factors, ILC1, ILC2 and ILC3 have been described with different functions for immunity and tissue integrity. While ILC1 are proinflammatory IFN-γ-producing cells (97), ILC2 play a role in atopic diseases and tissue repair (98), and ILC3 are important for tissue homeostasis and can inhibit pathological T cell responses (99, 100). Given their involvement in tissue repair and homeostasis, ILC are optimal cellular targets in GVHD, as tissue damage and impaired tissue homeostasis are the main factors in GVHD pathophysiology and drivers of the disease, in addition to autoreactive immune components (101).

Indeed, evidence from mouse models of GVHD suggests that ILC play a role in the pathogenesis of GVHD. Elegant mouse models showed that IL-23-responsive ILC3 produce IL-22, which protects intestinal cells from tissue damage and acts as critical regulator for GVHD (102). ILC-derived IL-22 reduced the severity of acute GVHD and GVHD-related mortality in this mouse model (102). A similar effect was shown in another mouse model with ILC2, which were severely affected by conditioning regimens (103). However, upon infusion of donor ILC2, gastrointestinal tract homeostasis was restored, Th1 and Th17 cells were reduced, while GVL was still preserved (103). Importantly, these effects were also observed upon adoptive transfer of ILC2 in mice with established GVHD, suggesting that ILC2 can be used not only to prevent GVHD, but also in a therapeutic setting (103).

In humans, correlations of ILC activation and incidence of GVHD in patients after allo-HSCT suggest that ILC recovery affects the development of GVHD (104). Furthermore, ILC3 are reduced in SR GVHD and can be restored by fecal microbiota transplantation correlating with the response to this experimental treatment (105). A subtle balance between proinflammatory Th17 and cytotoxic T cells on the one side and regulatory T cells and ILC3 on the other side seems to be important for the success of fecal microbiota transplantation (105). Besides their positive effect on tissue damage and restoring homeostasis, a subset of ILC3 expressing the ectoenzymes CD39 and CD73 can directly suppress T cell responses *via* the production of adenosine (106). This subset of ILC3 is depleted in patients with GVHD (106), which could be another factor contributing to the development of GVHD. As the presence of ILC3 in HCT grafts is associated with a reduced risk for GVHD (107), this cell population could be a candidate for adoptive transfer as GVHD-preventive or -therapeutic strategy.

Taken together, ILC2 and ILC3 positively affect GVHD and, together with other cellular targets, might represent a novel immune cell population to be utilized for therapeutic intervention.

## 6 Mesenchymal stromal cells

MSCs are fibroblast-like multipotent progenitor cells with immunosuppressive properties *in vitro* and *in vivo* (108). Although MSCs have extensively been used in clinical trials for GVHD therapy and have shown some efficacy, some studies did not reach statistical significance. Indeed, overall response rates of MSC for aGVHD range from 30-80% (109). This may be due to the fact that many trial patients had been heavily pre-treated, and due to different application procedures, doses, as well as production protocol (110). Furthermore, the source of MSCs may be critical for the therapeutic efficacy, as pre-clinical *in vivo* and *in vitro* studies have shown. Finally, the identification of adequate biomarkers could help to personalize and adapt effective cGVHD therapy or even prevention (111, 112).

### 6.1 Prophylactic use of MSCs in cGVHD

The prophylactic use of MSCs for cGVHD is summarized in a review by Morata-Tarifa et al. (113). Their meta-analysis confirms that the prophylactic administration of allogeneic bone marrow and umbilical cord MSCs can increase survival of pediatric and adult patients undergoing HSCT by reducing the incidence of cGVHD in 148 treated patients as compared to 236 controls. There was a significant heterogeneity in the overall survival across the individual studies indicating that there must be additional factors determining the survival rate (113). These could include the level of HLA-mismatch between donor and recipient (114), conditioning regimens (115), length and nature of immunosuppression (116, 117), as well as the number of repeated MSC doses (113). These diversifying factors given above may have caused a certain publication bias which can overcome by a standardization in the manufacturing process as well as adequately powered prospective studies. Both may help to confirm efficacy and safety of cGVHD treatment with MSC and thus improve the consistency of future clinical trials (118, 119).

### 6.2 Treatment of cGVHD with MSC

To our knowledge the first report on treatment of SR cGVHD with MSCs enclosed 19 patients (120). They received a median dose of bone marrow-derived MSCs from healthy donors with 0.6 x 10^6^ cells/kg. 73.3% of the patients responded well with 4 complete and 10 partial remissions whereas 5 patients died from relapse (n=2) and cGVHD-related complications (n=3). No patients experienced adverse events during or immediately after MSC administration. Interestingly, the clinical improvement was accompanied by an increased ratio of CD5^+^/CD5^-^ B cells and CD28^-^/CD28^+^ T cells, respectively, suggesting a shift to the more tolerant immunological phenotype. The authors conclude that MSC administration can be an effective salvage therapy for refractory cGVHD (120).

Recently, Li et al. reviewed and meta-analyzed 6 randomized and 13 non-randomized controlled trials comparing MSC co-transplantation in allo-HSCT with allo-HSCT alone (121). Generally, a co-infusion of MSCs improved engraftment and reduced the risk of cGVHD, notably less pronounced for aGVHD and non-relapse mortality. Specifically, the data obtained support the application of MSCs co-transplanted with HLA-nonidentical HSCT in children and young individuals (121). The data corroborates a previous study demonstrating that MSC therapy led to substantial improvements in terms of complete response and overall survival for cGVHD, with less influence, again, on aGVHD incidence, relapse or death (122).

MSCs may also work in patients with severe refractory cGVHD and still induce durable responses. In a recent study by Boberg et al. 11 patients received repeated infusions of allogeneic bone-marrow-derived MSCs over 6 to 12 months. 6 patients responded to MSC treatment following National Institutes of Health response criteria, accompanied by improvement in GVHD-related symptoms and quality of life. Of note, MSC treatment was associated with significant increases in naïve T cells, B cells, and Tregs 7 days after each infusion. Even prior to treatment responders had higher levels of naïve T and B cells. In addition, CXCL9 and CXCL10 chemokine levels were strongly elevated in responders as compared to non-responders, rendering them as potential new biomarkers of MSC therapy outcome (123).

An alternative mechanism of action how MSCs could benefit patients with cGVHD may be the induction of CD5^+^ regulatory B cells. In a prospective clinical study 23 refractory cGVHD patients were treated with 3^rd^ party bone marrow MSCs. 20/23 patients had a complete or partial response over a 12-month follow-up period. Clinical improvement was accompanied by a significantly increased number of IL-10-producing CD5^+^ B cells. Importantly, CD5^+^ B cells from cGVHD patients showed increased IL-10 expression after MSC treatment, which was associated with reduced inflammatory cytokine production by T cells (124).

It is well established that the vascular endothelium plays a pivotal role in the establishment of cGVHD and that endothelial cells can be targets of cGVHD-mediated immune responses (125, 126). There is *in vitro* evidence that some of these immune responses are not just allo-, but strictly endothelial-specific and cannot be controlled by the usual mechanisms, such as Tregs (127, 128). Allogeneic MSCs from different sources (bone marrow, umbilical cord, amniotic epithelium) have turned out to be protective against CD8^+^ endothelial-specific cytotoxic T cells (129), which may be another mechanism of action on how MSCs act.

A great deal of work has been spent on priming of MSCs to ameliorate their immunomodulatory/protective function. For example, pre-clinical data suggest that priming of MSCs with interferon gamma and hypoxia (130) or even with aGVHD and cGVHD-derived plasma (131) can significantly enhance MSC performance. Furthermore, in a humanized mouse model, umbilical cord-derived MSC primed with hypoxia and calcium ions attenuated GVHD significantly better than their naïve counterparts (132). It remains to be elucidated if this approach will also work for clinical cGVHD.

In conclusion, MSCs generally qualify for prophylaxis and/or treatment of refractory cGVHD, but larger randomised controlled trials with repeated doses of cells accompanied by well-designed biomarker studies will be necessary to further advance this therapeutic option.

### 6.3 MSC-derived extracellular vesicles

Despite their beneficial properties as a cellular therapy for aGVHD, MSCs have demonstrated a lack of correlation between functional improvement and engraftment as well as differentiation at the site of tissue damage or injury. Many hypotheses now center around the belief that it is not the MSCs themselves, but rather their secretome, including extracellular vesicles (EVs) that are driving their therapeutic efficacy. Thus, known as the ‘paracrine hypothesis’. Indeed, co-culture of MSCs with Tconvs in a non-direct transwell system results in Treg induction (133). The potency attainable by MSC may also be inferior to MSC-EV. For instance, MSC suppressed T cell expansion to a lesser extent than MSC-EVs derived from the same number of MSC cells (134). Overall, MSC-EV have been shown to exert an inhibitory effect on T-cell activation and differentiation, as well as reducing T-cell proliferation and IFN-γ release (135). Although the full beneficial use of MSC-EV as a GVHD therapeutic is still being explored, several studies are indicating promising results.

**Figure 3:**
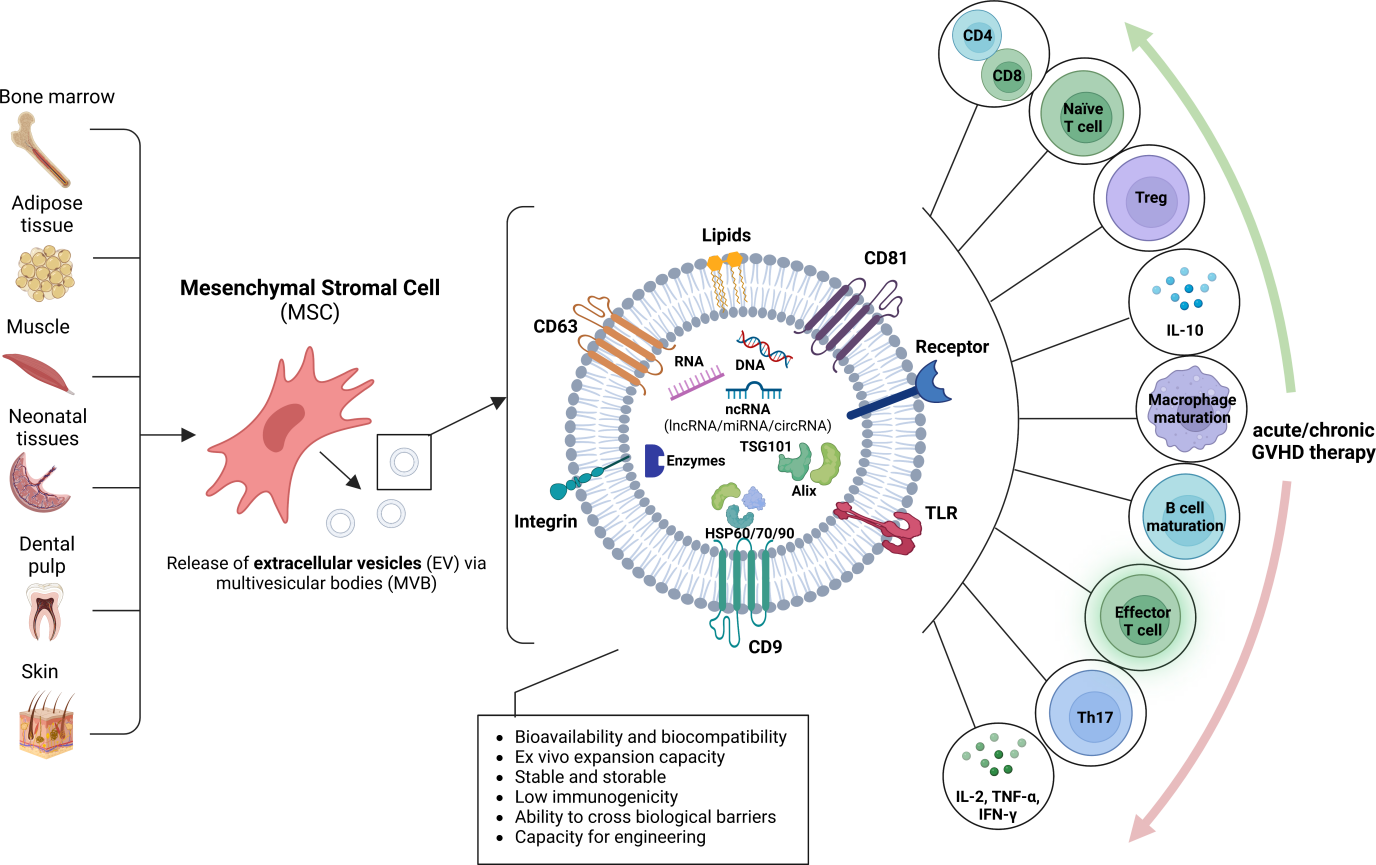
Origin of mesenchymal stromal cell extracellular vesicles and putative role for GVHD. Mesenchymal stromal cells (MSC) may be derived from a variety of sources, including bone marrow, adipose tissue, muscle, neonatal tissues, dental pulp and skin. The MSC release extracellular vesicles (EV) by inward budding of the plasma membrane and formation of intracellular multivesicular bodies (MVB), followed by exocytosis. MSC-derived EVs are enriched in various proteins with multiple functions, such as biogenesis-related proteins (e.g., TSG101, ALIX), common surface markers (e.g., CD9, CD81, CD29, CD44 and CD90), membrane transporter and fusion proteins (e.g., Rab GTPases and annexins), integrins, heat shock proteins (e.g., HSP60, HSP70 and HSP90) and MHC class I and II proteins, DNA, RNA and noncoding RNA (ncRNA), including miRNA, lncRNA, and circRNA. The MSC-EVs may modulate acute or chronic GVHD by acting on diverse immune cells.

MSCs have been shown to secrete the main subcategories of EV, including microvesicles (136), microparticles (137) and exosomes (138). MSC-derived EVs are enriched in bioactive molecules including lipids, proteins, mRNA, tRNA, lncRNA, microRNA and mitochondrial DNA (139) and have been proposed to interact with heterogeneous cell types as a communication vesicle to maintain a dynamic and homeostatic tissue environment (140) (**Figure 3**).

MSC-EV demonstrate great potential as therapeutic agents, as they carry a number of advantages over their parental cells. From a practical perspective, MSC-EV can be easily isolated from healthy adult tissues and they have great *ex vivo* expansion capacity. Their small size and robust membrane makes them easier to handle than cells, and freezing, thawing and storage conditions are less critical (141). Furthermore, as they are produced from cell supernatants and not the cells themselves, large scale production is more feasible (141).

MSC-EV are less likely to trigger an adverse immune response compared to their parental cells, due to their lack of major histocompatibility complex class I/II molecules, which makes them attractive as a safer therapy (142). Indeed, they have proven to be safe in both human and animal models, with no observed side effects (143, 144). As MSC-EV are nano-sized in nature, they can migrate through most physiological barriers, allowing effective concentrations to accumulate in target tissues (145). As EVs are non-viable and non-replicating they may avoid the risk of unregulated cell growth, autoimmune disease and occlusion in the microvasculature (145, 146). Furthermore, the biological activity and functional properties of MSC-EV may be more precisely defined compared to their parental MSCs, as they lack complex metabolic activity and as such, the risk of reprogramming by the environment is reduced (141).

The nano-size of MSC-EV allows for sterilization by filtration. This should effectively minimize the risk of biological contamination, and accordingly, the regulatory requirements for clinical grade production of EVs may not be as restrictive as for cellular therapy (147). Indeed, since MSC-EV from conventional MSC do not contain a nucleus or transgenic product they do not fall into a currently defined advanced therapy medicinal product (ATMP) category. They are therefore exempt from regulations under the European Medicines Agency (EMA), and guidelines for their standardized production and quality assurance are not yet defined.

However, the use of MSC-EV comes with some caveats. To date, recommendations concerning the production and application of EV-based therapies have been advised by the International Society for Extracellular Vesicles (ISEV), but these are simply guidelines and not currently regulated (148). Although MSC-EVs possess potent immunomodulatory properties, their immunosuppressive capacity is not constitutive. In addition, there is no standardization surrounding the optimal protocols for isolation of MSC-EV and identifying or characterizing MSC-EV phenotypes (145). Much work is required between researchers, clinicians and the regulatory authorities in order to stand arise all aspects relating to production of EV-based therapeutics prior to routine clinical application, including but not limited to the source of the starting material, EV isolation and storage methods, quality control aspects and *in vivo* analyses/EV application, as summarized in an eloquent position paper by the International Society for Extracellular Vesicles (ISEV) (141).

### 6.4 MSC-EVs for treatment of cGvHD

MSC-EVs are immunologically active and induce elevated expression of anti-inflammatory *IL10* and *TGFβ1*, and reduced levels of pro-inflammatory *IL1β, IL6, TNFA* and *IL12P40*. Furthermore, they can induce Tregs both *in vitro* and *in vivo*, and MSC-EV infusion has been shown to enhance the survival of allogeneic skin grafts in mice (149). Studies by Zhang et al. demonstrated that the immunosuppressive activities of MSC-EVs are mediated in part by activation of MYD88-dependant signaling in monocytes to induce an anti-inflammatory M2-like phenotype *via* a TLR-dependent signaling pathway (149). Activated monocytes then polarize activated Tconvs to Tregs, inducing Treg expansion and an attenuated activated immune system (149).

MSC-EVs have been shown to replicate the therapeutic effects of MSC cells in models of acute lung injury, skin wounds and myocardial ischemia (150, 151). Although studies relating to the therapeutic effect of MSC-EV in cGVHD are in their infancy, MSC-EV have been demonstrated as a promising therapeutic tool for treating pulmonary complications of cGVHD (145) and exhibit potent suppression of autoreactive T cell activation and migration, which is essential in the pathogenesis of cGVHD (145).

In their seminal 2014 study, Kordelas et al. administered MSC derived EVs to a SR GVHD patient, with the hypothesis that MSC-EVs may offer improved outcome compared to MSC therapy (143). The MSC-EV fractions from four unrelated bone marrow donors were tested for their effect on patient derived PBMC cytokine secretion. MSC-EV fraction 1 contained the highest content of anti-inflammatory molecules, and a mixed lymphocyte reaction (MLR) with patient PBMC’s resulted in immunosuppressive action by reducing IL-1β, TNF-α and IFN-ɣ expression (143). Following patient administration, all applications were tolerated well with no detected side effects. During the course of MSC-EV therapy, the patient’s pro-inflammatory cytokine response were reduced (IL-1β, TNF-α and IFN-ɣ) and the clinical symptoms of GVHD improved, including indications of cutaneous and mucosal GVHD, which was stable 4 months following completion of therapy (147).

In a follow up clinical case report, Norooznezhad et al. used human placental MSC (hPMSC)-derived EVs to treat a patient with cutaneous cGVHD that was unresponsive to ECP and high-dose corticosterioids (152). The patient received 4 treatments at a weekly interval, comprising of 1.9-2.6 x 10^11^ EV particles administered in saline, and treatment was well tolerated with no side-effects observed. 15 days-post treatment, skin hyperpigmentation was reduced and the frequency and severity of ulcers, wounds and keratotic lesions was decreased. Monocyte levels were also significantly reduced from 18% to 5%, and clinical changes were sustained for 5 months of follow up assessment (152). This study further highlights the potential of MSC-EV therapy for cGVHD and strengthens the need for follow up larger scale trials, as well as investigation of the impact of MSC source for EV harvesting.

In mouse model pre-clinical studies to assess mechanisms of MSC-EV action, Lai et al. employed a cGVHD mouse model and tail vein injected bone marrow derived MSC-EV on day 22 following BMT (145). The EV injections were administered once per week for 6 weeks and test mice were compared to control mice injected with human dermal fibroblast EVs, or blank control mice injected with equal volumes of PBS (145). They observed that MSC-EV treatment effectively prolonged the survival of cGVHD mice and diminished the clinical and pathological cGVHD scores (145). The activation and lung infiltration of CD4^+^ T cells was reduced, and skin, lung and liver fibrosis was ameliorated (145). The potent immunomodulatory effects observed were shown to be *via* inhibition of IL-17-expressing pathogenic T cells and induction of IL-10 regulatory cells (145). By 35 days post-BMT, the MSC-EV mice demonstrated few clinical GVHD features, low disease and skin scores of cGVHD pathology and significantly improved survival compared to control mice, which demonstrated characteristic clinical signs of cGVHD, suggesting MSC-EV to have an inhibitory effect on cGVHD. The activation and infiltration of CD4^+^ T cells in target organs was also inhibited, and CCR6 expression was reduced, which normally functions to recruit Th17 cells (145). Reduced Th17 frequency and upregulated IL-10 and FOXP3 was also observed in the splenocytes and lymph nodes of MSC-EV treated mice (145). Lai et al. also assessed the effect of MSC-EV on pro-inflammatory cytokine production and observed significantly lower levels of IL-17A, IL-22 and IL-21 and IL-2 in MSC-EV treated mice, while IL-10 levels were increased 2-fold (145). Importantly, the effect of MSC-EV on human PBMCs was tested *in vitro*. The authors showed that MSC-EV are taken up by CD3 cells and upregulate the percentage of CD25^+^Foxp3^+^CD4^+^ Treg in PBMCs from normal donors. When the same PBMCs were cultured under Th17 conditions, MSC-EV suppressed Th17 differentiation. In PBMCs from patients with active GVHD, IL-17 expressing CD4^+^ T cells were reduced and IL-10 production was increased upon MSC-EV treatment. Finally, MSC-EV suppressed expression of RORɣt and Stat3, while upregulating production of Foxp3 (145). These important findings indicate that MSC-EV’s therapeutic action in cGVHD may be attributed to expansion of Tregs while inhibiting pro-inflammatory Th17 cells, suppressing migration and infiltration of CD4^+^ T cells into target organs. The MSC-EV also exert immunosuppressive effects on cytokine production (145). However, despite these informative results, the group did not explore the molecular cargo of the MSC-EV in order to fully elucidate their immunomodulatory effects in the mouse model of cGVHD studied, or the Th17/Treg differentiation modulation observed.

In a further pre-clinical study, Fujii et al. also investigated the mechanisms by which MSC-EV may ameliorate GVHD-associated complications, based on an acute model of disease (134). They used MSC-EVs from the bone marrow of healthy volunteers and assessed the effect of EVs on functional T cell subsets *in vitro.* In the presence of MSC-EVs, analysis of peripheral blood T cell revealed suppression of CD8^+^ T cell expansion, a decreased frequency of effector T cells and increased frequency and number of naïve T cells, suggesting an overall suppression of functional differentiation of T cells from a naïve to effector phenotype (134). MSC-EV suppressed T cell expansion, while B cell, NK cell and mature myeloid cell populations were not affected. The MSC-EV treated mice demonstrated prolonged survival, and mitigated GVHD-associated pathology in the skin and large bowel. Interestingly, authors performed some limited assessment of the MSC-EV cargo and microarray analysis identified 336 microRNAs that were upregulated and 337 microRNAs that were downregulated compared to normal human dermal fibroblast EVs (NHDF-EV), including miR-125a-3p which was the most highly upregulated microRNA in MSC-EV (134). GO enrichment analysis revealed the most highly up and down-regulated microRNAs in MSC-EVs were predicted to target proliferation-related genes, while KEGG pathway analysis revealed genes with a role in cell cycle regulation, T cell receptor signalling and GVHD.

In a pre-clinical model to investigate prevention of GVHD, Zhang et al. showed that MSC-EVs can replicate the paracrine potency of MSCs in generating Tregs, in a capacity mediated by antigen presenting cells (146). This was corroborated in a chimeric hu-SCID mouse of GVHD, whereby GVHD symptoms and survival were improved by MSC-EV treatment, with surviving mice demonstrating higher Treg levels in both the blood and spleen (146). Wang et al. demonstrated that human umbilical cord-derived EVs (hUC-MSC-EVs) had the capacity to act prophylactically against aGVHD following intravenous administration to a mouse model of allo-HSCT on days 0 and 7 post-HSCT (153). Indeed, treated mice showed significantly lower frequencies and absolute numbers of CD8^+^ T cells, reduced serum IL-2, TNF-α and IFN-γ levels, a higher ratio of CD4^+^ and CD8^+^ T cells and higher serum IL-10 levels, strengthening the prophylactic use of MSC-EV in order to modulate immune responses. Overall, the severity of GVHD manifestations were reduced at day 28 post-HSCT, with reduced weight loss, improved GVHD score, prolonged survival and reduced histology scores for GVHD-associated changes (153).

Despite these numerous reports highlighting the beneficial potential of MSC-EV for GVHD prevention and treatment, as well as their role in informing on the pathophysiology of GVHD, use of MSC-EV prevents several caveats. Indeed, the field of EVs is still in its infancy, for which several Task Force groups within the International Society for Extracellular Vesicles are working to address fundamental issues supporting their use. Many of these issues are comprehensively described elsewhere (141, 148, 154, 155) and are beyond the scope of this review, but when considering MSC-EV for therapy, special attention should be given to factors such as MSC source (e.g bone marrow, adipose tissue, synovial membrane, umbilical cord), EV production (eg. culture system, medium composition, cell-adherence support, bioreactors, stimulation), EV isolation (e.g. centrifugation techniques, size-based fractionation, ultrafiltration), quality controls, EV dosage and storage, and stability. Thus, the heterogeneity of MSCs used for EV production as well as of the isolated EVs requires extensive further consideration and will be the focus of researchers, clinicians and regulatory authorities prior to any approved industrial or clinical use of MSC-EV.

## 7 Safety aspects associated with cellular therapy

Cell and gene therapy ATMPs are extensively regulated by a number of regulatory bodies world-wide. These include the US Food and Drug Administration (FDA) and EMA. The FDA and EMA both interact *via* the International Council for Harmonisation of Technical Requirements for Pharmaceuticals for Human Use (ICH) with the aim to ensure adequate guidelines and procedures for the development of advanced investigational medicinal products for clinical trial use. Apart from the manufacturing safety considerations, which include batch consistency, detection of impurities, specificity and purity of the product, specific safety aspects associated with each type of ATMP needs to be considered.

Within this review we have discussed T cells, NK cells, iNKT cells, ILCs, and mesenchymal cells. Although some of these cellular therapies have very good safety indications, such as Tregs, anti-viral T cell products, NK cells, and MSC-EV, other therapies such as CAR-T cells have been associated with bystander effects such as cytokine release syndrome, graft versus host disease (if an allogeneic product) and neurotoxicity. Consideration therefore needs to be given to the development of novel human based *in vitro* models to predict and understand some of these safety issues prior to clinical trial development.

The current guidelines from EMA, FDA and ICH detail safety requirements for clinical trials based on that ensuring patient safety and clear risk assessment strategies are well documented^1^. Although immunogenicity risks are clearly defined for pharmaceuticals, the types of *in vitro* testing requirements are less clear within the guidelines and not specified especially with regard to cellular therapies. There is a current consensus that *in vitro* testing using human cells or tissues, if validated and have clear indications of predicting clinical outcome, should be used in preference to animal model experimentation. This has been further advocated by the passing of the FDA Modernization Act of 2021 in June 2022 in the US House^2^, ending the outdated mandate that all drugs must be tested on animals for registration dossiers. More research is needed, for example to develop assays for the more complex toxicities, such as neurotoxicity which will need collaborations between both and commercial and academic groups in order to achieve safe and effective ATMP development in the future.

## Author contributions

MD, RC, OP, AA, AD, GS, JL, GE, and MI planned contents and wrote the manuscript. All authors contributed to the article and approved the submitted version.

## Funding

This work was supported by COST (European Cooperation in Science and Technology). www.cost.eu - COST Action 17138 EUROGRAFT.

## Conflict of interest

AD was employed by Alcyomics Ltd.

The remaining authors declare that the research was conducted in the absence of any commercial or financial relationships that could be construed as a potential conflict of interest.

## Publisher’s note

All claims expressed in this article are solely those of the authors and do not necessarily represent those of their affiliated organizations, or those of the publisher, the editors and the reviewers. Any product that may be evaluated in this article, or claim that may be made by its manufacturer, is not guaranteed or endorsed by the publisher.

## Footnotes

1
https://www.ema.europa.eu/en/documents/scientific-guideline/guideline-strategies-identify-mitigate-risks-first-human-early-clinical-trials-investigational_en.pdf.

2
https://www.congress.gov/117/bills/hr2565/BILLS-117hr2565ih.xml.
